# Darwin's *Other* Books: “Red” and “Transmutation” Notebooks, “Sketch,” “Essay,” and *Natural Selection*

**DOI:** 10.1371/journal.pbio.0030382

**Published:** 2005-11-15

**Authors:** Niles Eldredge

## Abstract

Study of Darwin's unpublished works, freely available on-line through the American Natural History Museum, reveals the origins of his thoughts on evolution.

[Fig pbio-0030382-g001]

**Figure pbio-0030382-g001:**
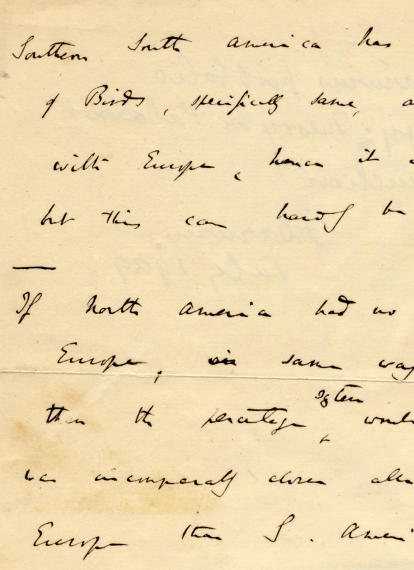


Depending on how you count them up, Charles Darwin published just over twenty books in his lifetime. His first—the *Journal of Researches* [1], also known as *The Voyage of the Beagle* was his most famous—until Darwin, pressured by the arrival in 1858 of A. R. Wallace's manuscript on evolution through natural selection, stopped working on his “Big Species Book,” [2] and wrote instead his epochal *On the Origin of Species by Means of Natural Selection. Or the Preservation of Favoured Races in the Struggle for Life* [3]. In between, and thereafter, Darwin published monographs and specialized narratives on topics as disparate as barnacle taxonomy, coral reef development, and insectivorous plants. Yet it is, of course, the *Origin of Species* that changed the world, establishing Darwin as one of the great thinkers in Western cultural history.

So much is well-known. Far less appreciated is the fact that Darwin wrote several other books, all on evolution, none of which were published in his lifetime. Together, they form a series that preserves the “evolutionary” history of Darwin's ideas from their very inception to their most mature form—while also revealing the more prosaic development of Darwin's written rhetoric of the *Origin of Species*. All have been subsequently published, and are now freely available online, constituting the initial components of the Darwin manuscript project page of the American Museum of Natural History Digital Library of Evolution. The Darwin manuscript project complements the museum's exhibition, Darwin, opening 19 November 2005; further analysis of these works can be found in my companion volume to the exhibition [4].

The first of these books is actually a series of notebooks. I include them because they are the foundational writings for all Darwin's later, more discursive, discussions of evolution. Many of the themes—and, indeed, some of the original language—of the familiar passages of the *Origin of Species* are found in the pages of these notebooks—maddeningly interspersed in near-chaotic fashion with all manner of geological and biological observations and notations gleaned from the literature, and Darwin's already burgeoning correspondence and conversations. Indeed, only the “principle of divergence” came along later to add to Darwin's themes and arguments.

## “Red” and “Transmutation” Notebooks

The “Red Notebook” [5] is the first of the series of notebooks in which Darwin established the essential elements of his evolutionary theory. Although apparently started while still on the *Beagle* in 1836—recording various latitude, longitude, and depth soundings—the last third of the notebook seems to have been filled out after Darwin returned to England in late 1836, early 1837. Historians still disagree whether or not—or the degree to which—Darwin had tumbled to the idea of evolution while still on the *Beagle*. I fully agree with Kohn et al. [6] that the famous passage in his *Ornithological Notes*, discussing the differentiation of “varieties” of mockingbirds and tortoises on various islands in the Galapagos and concluding that “if there is the slightest foundation for these remarks to zoology of Archipelagoes—will be well worth examining; for such facts would undermine the stability of Species” [7] in fact does establish that Darwin was thinking about evolution in the final months before the *Beagle* arrived home. But nothing else unambiguously written while still aboard ship has as yet turned up to support this view.

Darwin was a fully committed evolutionist by the time the evolutionary passages of the “Red Notebook” were written. As he would subsequently write in the topic sentence of the *Origin of Species*, Darwin had been greatly struck by “certain facts of the distribution of the inhabitants of South America” that “throw some light on the origin of species.” [3]. Elsewhere, Darwin makes clear that there were three distinct patterns of replacement of “allied” forms. First, the replacement of extinct species by modern ones—belonging to groups unique to that part of the world. For example, armadillos now live, while the obviously closely similar giant glyptodonts (which Darwin collected in Argentina) are now extinct. Both are edentate mammals—found only in the Americas. Second, in the living world, closely similar species tend to replace each other over broad expanses of mainland South America. The original example of this is the replacement of the common rhea (ostrich-like bird) by the lesser (Darwin's) rhea in more southerly stretches of South America. And third, the replacement by similar varieties or species of animals and plants on different islands (in the Galapagos especially—but he also mentions the two different forms of fox found on each of the two Falkland Islands). The mockingbirds and tortoises are early examples; as is well-known, Darwin did not himself see similar replacement patterns in finches, which later became known as “Darwin's finches,” and the equally riveting plant examples had to await expert analysis, forthcoming only after Darwin had been home for some time.

Darwin, famous for his views of gradual evolution through natural selection in the *Origin of Species*, is unexpectedly a saltationist in the “Red Notebook.” He thinks, given the lack of intergradations between fossil forms, or his rheas, that new species must arise suddenly from ancestral species. He maintains this view to some degree in Notebook B, first of the four famous “Transmutation Notebooks” [8], begun in the summer of 1837 and finished in early 1838. But with Notebook B, his attention turns to defining the first of three additional patterns, seeing these as expected observations if evolution is true. Darwin's initial three replacement patterns were inductive generations that took some while to dawn on his conscious mind. Now, with Notebook B he turns the tables and establishes the idea of evolution in a hypothetico-deductive framework.

There is grandeur in this view of life.

First of these new expected patterns is the nested set of taxa already recognized and embodied in Linnaeus's *Systema Naturae* [9]. We now know why, in other words, there seems to be a natural classification of species—an explanation that differs from creationism precisely because it does make predictions about what we should expect to observe if evolution is true. In what is Darwin's closest equivalent to Einstein's handwritten E = MC^2^, he writes (Notebook B, page 36) “I think,” [8] and sketches an abstract evolutionary tree. He goes on to add embryological resemblance and “the unity of type” (homology), all close correlates of the “natural system”—all seen as predicted observations under the theory of transmutation (descent with modification eventually equals evolution).

But Darwin wanted more: he was constantly searching for a mechanism. Finally, in Notebook D, after having read Thomas Malthus and learned for the first time that more organisms are born to each species each generation than can possibly survive and reproduce (otherwise, “the world would be standing room only in elephants after but a few thousand years,” [3] he wrote later in the *Origin of Species*), he formulated “natural selection.” As David Kohn [10] first pointed out, Darwin parses natural selection pithily on page 58 of Notebook E (1839): “Three principles will account for all: (1) Grandchildren like grandfathers (2) Tendency to small change «especially with physical change≫ (3) Great fertility in proportion to support of parents” [8]. In other words, (1) heredity, (2) variation (Darwin thought variation was induced in large measure spontaneously in the reproductive process and by the environment—views he held throughout his writings), and (3) the Malthusian principle of overproduction.

Therefore, natural selection—though not called such until the next “book” in our series (the 1842 “Sketch”). Darwin had been using the expression “my theory” to mean “evolution.” But now, the expression “my theory” more specifically means “evolution by natural selection.” It is in 1839, toward the end of the series of “Transmutation Notebooks,” that Darwin takes his next logical, if not fateful, step: in page 118 of Notebook E, he exhorts himself to rederive his original patterns in terms of his ideas on how natural selection works to produce evolutionary change. He is by now far beyond his initial attraction to saltational evolution: rather natural selection must produce finely gradational change. This puts him at odds with his very first evolutionary pattern, as Darwin is aware that paleontologists see little evidence of such change in their collections of fossilized plants and animals. He writes (Notebook E, page 6): “My very theory requires each form to have lasted for its time: but we ought in same bed if very thick to find some change in upper & lower layers.—good objection to *my theory*: a modern bed at present might be very thick & yet have same fossils” [8] Darwin, an intellectually very honest man, was troubled by this “good objection” throughout his evolutionary writings—devoting a chapter to the problem and essentially inventing the science of taphonomy (study of the formation of the fossil record) in *Origin of Species*.

After discovering natural selection in Notebooks D and E, Darwin turns renewed attention to both variation and the process of artificial selection in embryonic form— the analogy to what he would soon call “natural selection” by this time clear. The theme that varieties are incipient species—perhaps the most pervasive of Darwinian argumentative themes—is found in these notebooks [6], as are some other, more rhetorical, devices that show up in the later works, including the *Origin of Species*.

Particularly striking is Darwin's invocation of the travails of astronomers who labored so hard (occasionally relinquishing their lives!) to establish the laws of gravitation governing the behavior of celestial bodies. Darwin was not only fearful of attack on religious grounds, he also knew all too well that the only competing theory to explain the origin and diversity of life was in fact Judeo–Christian creationism. In Notebook B (page 101) [8], Darwin writes: “Astronomers might formerly have said that God ordered each planet to move in its particular destiny—in same manner God orders each animal created with certain form in certain country, but how much more simple and sublime power let attraction act according to certain laws such are as inevitable consequence let animal be created, then by the fixed laws of generation, such will be their successors—let the powers of transportal be such & so will be the form of one country to another—let geological changes go at such a rate, so will be the numbers & distribution of the species!!” Later in the notebooks, he mentions persecution of the astronomers—and also writes (Notebook D, page 36): “What a magnificent view one can take of the world Astronomical <& unknown> causes, modified by unknown ones. cause changes in geography & changes of climate superadded to change of climate from physical causes.—these superinduce changes of form in the organic world, as adaptation. & these changing affect each other, & their bodies, by certain laws of harmony keep perfect in these themselves.—instincts alter, reason is formed, & the world peopled with Myriads of distinct forms from a period short of eternity to the present time, to the future—How far grander than idea from cramped imagination that God created (warring against those very laws he established in all organic nature) the Rhinoceros of Java & Sumatra, that since the time of the Silurian, he has made a long succession of vile Molluscous animals—How beneath the dignity of him, who is supposed to have said let there be light and there was light…” [8].

This passage is “ancestral” to Darwin's most famous passage—concluding the *Origin of Species* some 21 years later. Here we have not only the analogy with scientific law replacing creationist belief in astronomy, but also the origin of the famous phrase “there is grandeur in this view of life” [3,11,12]. We even see here reference (albeit only in passing) to Javan and Sumatran rhinos—expanded and integral to the conclusions of each of Darwin's successive books on evolution.

## The 1842 “Sketch” and 1844 “Essay”

In 1909, Francis Darwin (Charles and Emma's seventh child), on the 100th anniversary of his father's birth, published *Foundations of the Origin of Species* [13]. The book contained Francis's transcription of two of his father's unpublished, handwritten manuscripts, the “Sketch” of 1842 [11] and the much longer, discursive, and on the whole better-written “Essay” of 1844 [12].

The “Sketch” is Darwin's earliest known (and almost undoubtedly his very first) attempt to write out his evolutionary theory in essay form. The fact that it was never intended for publication, but rather served as a first-shot “dry-run” in setting out his views, is amply demonstrated by the sometimes elliptical, almost notebook-like passages with incomplete sentences and occasional reminders to himself on how to develop his arguments further. (Indeed, the last two paragraphs of Francis's 53-page edition are just these sorts of notes to himself).

The 1842 “Sketch” is an exciting read. Darwin is effectively organizing his thoughts and putting them in more coherent form for the first time. He adopts a two-part structure (retained in his 1844 “Essay,” with part 1 (three chapters) a succinct statement of his theory of the mechanisms of evolution, and a longer part 2 (seven chapters) the application of his ideas of evolution through natural selection to the, by now, familiar patterns of the biological world (geographic replacement, classification, embryology, unity of type (homology)—and the persistent problems with the fossil record).

It is in the second chapter of part 1 that we see the fateful two words “natural selection” as a subhead of a section that lays out by far his most coherent description of the process to date: “DeCandolle's war of nature—seeing contented face of nature—may be well at first doubted; we see it on borders of perpetual cold. But considering the enormous geometrical increase in every organism and as every country, in ordinary cases, must be stocked to full extent, reflection will show that this is the case. Malthus on man—in animals no moral restraint—they breed in time of year when provision most abundant, or season most favourable….The unavoidable effect of this is that many of every species are destroyed either in egg or young or mature….In the course of a thousand generations infinitesimally small differences must inevitably tell….Nature's variation far less, but such selection far more rigid and scrutinizing” [11].

The bulk of Darwin's 1842 text integrates all he has read in books, monographs, and correspondence about variation, artificial selection, patterns of geographic distribution of animals and plants, and gradation between varieties and distinct species—the main topics of his notebooks. He continues the hypothetico-deductive theme begun in Notebook B—showing that such patterns should be expected as the natural outcome of the evolutionary process.

His “Recapitulation and Conclusion” in the 1842 “Sketch” is a brilliant, impassioned summary of his ideas. It ends with the passage, already adumbrated in Notebook D, that remains virtually identical, not only in 1844 but in the *Origin of Species* itself: “There is a simple grandeur in the view of life with its powers of growth, assimilation, and reproduction, being originally breathed into matter under one or a few forms, and that whilst this our planet has gone circling on according to fixed laws, and land and water, in a cycle of change, have gone on replacing each other, that from so simple an origin, through the process of gradual selection of infinitesimal changes, endless forms most beautiful and most wonderful have been evolved” [11].

For the most part, Darwin's fervent intellectual search is over with the conclusion of the 1842 “Sketch.” The 1844 “Essay,” at 198 pages, is a much longer manuscript; with exactly the same structure and sequence of topics, it is essentially a smoothed out version of its predecessor—written completely in essay form now, all sentences complete, with no personal notes and queries to interrupt the flow of ideas. The bulk of it, for the most part, consists of vastly more examples bolstering Darwin's points throughout.

That said, the 1844 version of his ideas is far less exciting to read than the 1842 manuscript. It is very much as if the excitement is muted by the sheer bulk of the material reviewed—and probably as much by the fact that the ideas are no longer so novel to Darwin himself. Freshness is lost to familiarity and the sheer weightiness of his verbiage.

## *Natural Selection* and *On the Origin of Species*

Much the same can be said of Darwin's so-called abstract of his views—*The Origin of Species*. This, his most famous book, was fresh and new to its readers in 1859, so successful had Darwin been in keeping his views private. But to anyone who has had the privilege of reading the 1830s notebooks, and the early manuscripts (especially 1842), the *Origin of Species* reads like a mature work in both the best and worst sense of the term. He has honed his arguments beautifully, but the ideas, no longer fresh in his own mind, are just not as enthrallingly expressed as when he was younger and much closer to their inception.

Darwin's Principle of Divergence—poorly understood by modern scholars—melds nascent ecological theory with various models of the origination of new species. Darwin was developing those ideas around the time he started writing his “Big Species Book,” eventually published (second part only) as *Natural Selection* [2]. And though one shudders at the sheer voluminous nature of this gigantic, yet partial, book, and is tempted to be glad that Darwin pared it down to the more manageably sized *Origin of Species*, it is true that, at least as far as the discussion of the Principle of Divergence is concerned, the discussion in this last of Darwin's unpublished-in-his-lifetime books is more cogent and complete than the *Origin of Species* itself. The *Origin of Species*, one is tempted to conclude, written as it was in such haste, relied heavily on the earlier manuscripts. (Darwin dispensed with the two-part structure, but maintained the same basic sequence of chapters and topics.) In many ways, the unpublished versions hold more rewards than what the *Origin of Species*—the book that shook the world—offers the modern reader.

There is much more that can be said. Darwin's thoughts about the relative importance of isolation, for example, changed over the years. It is possible with this treasure trove of Darwin's *other* books to pick themes and trace their development over time. Seldom has the history of ideas, so important as Darwin's evolution by natural selection, been so faithfully preserved as it has in this virtual “fossil record” preserved in this magnificent series of Darwin's *other* books.

The "Red" and "Transmutation" notebooks (1836-1839), the "Sketch" (1842), the "Essay" (1844), and *Natural Selection* (1856-1858) are freely available online at http://darwinlibrary.amnh.org
